# Tuberactinomycin antibiotics: Biosynthesis, anti-mycobacterial action, and mechanisms of resistance

**DOI:** 10.3389/fmicb.2022.961921

**Published:** 2022-08-11

**Authors:** Zane T. Laughlin, Graeme L. Conn

**Affiliations:** ^1^Department of Biochemistry, Emory University School of Medicine, Atlanta, GA, United States; ^2^Graduate Program in Biochemistry, Cell and Developmental Biology (BCDB), Graduate Division of Biological and Biomedical Sciences, Emory University, Atlanta, GA, United States; ^3^Emory Antibiotic Resistance Center (ARC), Emory University, Atlanta, GA, United States

**Keywords:** 70S ribosome, antibiotic resistance, mycobacteria, capreomycin, viomycin, methyltransferase, tuberculosis antibiotics, rRNA modification

## Abstract

The tuberactinomycins are a family of cyclic peptide ribosome-targeting antibiotics with a long history of use as essential second-line treatments for drug-resistant tuberculosis. Beginning with the identification of viomycin in the early 1950s, this mini-review briefly describes tuberactinomycin structures and biosynthesis, as well as their past and present application in the treatment of tuberculosis caused by infection with *Mycobacterium tuberculosis*. More recent studies are also discussed that have revealed details of tuberactinomycin action on the ribosome as well as resistance mechanisms that have emerged since their introduction into the clinic. Finally, future applications of these drugs are considered in the context of their recent removal from the World Health Organization’s List of Essential Medicines.

## Introduction

**Figure 1:**
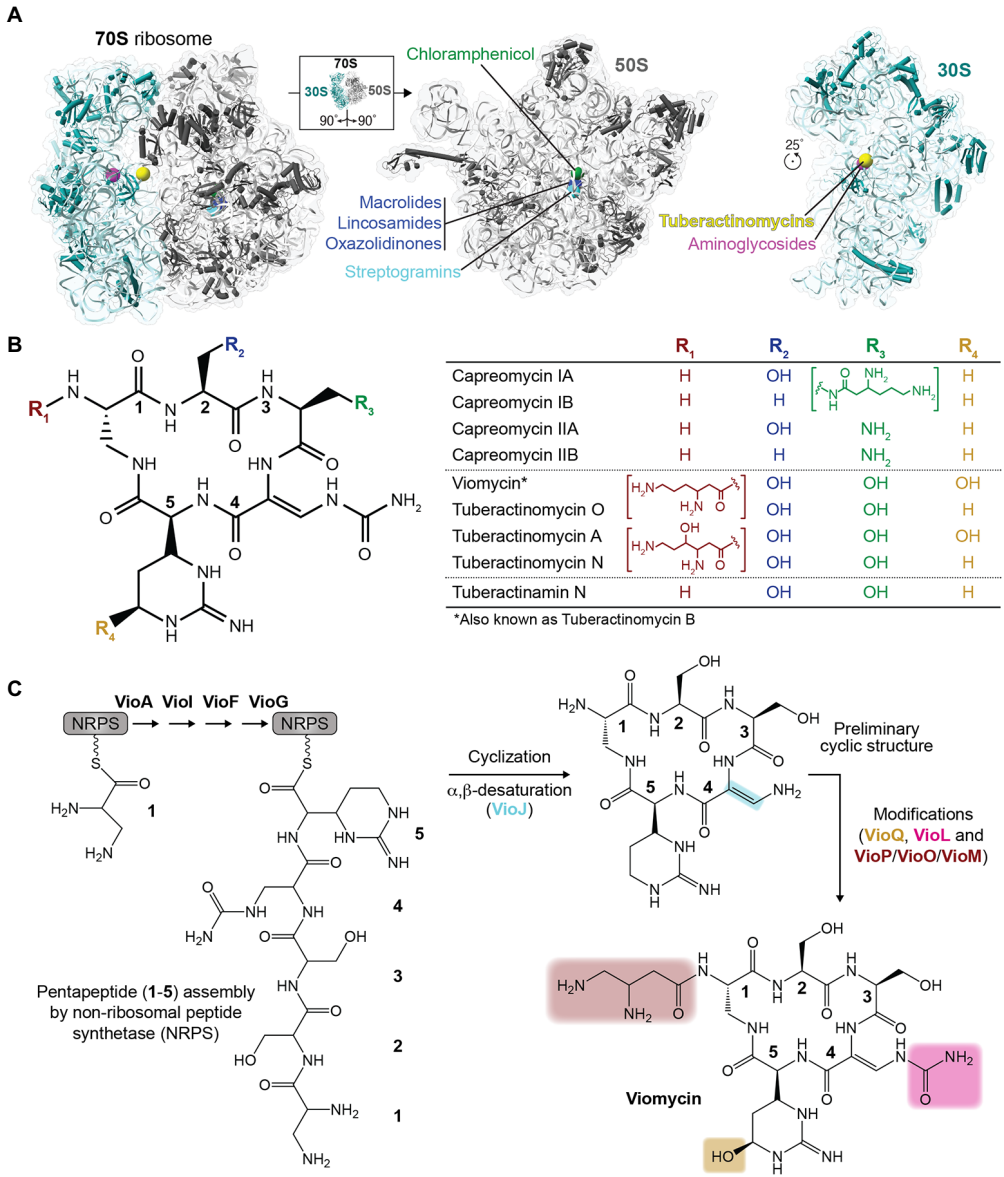
Ribosome-targeting antibiotics and chemical structures of the tuberactinomycins. **(A)** Sites of action of select clinically relevant ribosome-targeting antibiotics are shown on the structure of the intact *Escherichia coli* 70S ribosome (*left*) and individual subunits (right) rotated as indicated to show their subunit interfaces (PDB code 6LKQ). The indicated antibiotics target the 50S (large) subunit at the peptidyl transferase center and nascent peptide exit tunnel (chloramphenicol, lincosamides, oxazolidinones, streptogramins, and macrolides), or the 30S (small) subunit decoding center and nearby inter-subunit interface (aminoglycosides and tuberactinomycins). **(B)** Chemical structures of the tuberactinomycin antibiotics shown as the common pentapeptide core scaffold (*left*) and unique substituents at four variable positions (R1 to R4; as indicated in the table on the *right*). **(C)** Overview of the process of tuberactinomycin biosynthesis (shown for viomycin), including non-ribosomal peptide synthetase (NRPS) assembly of the pentapeptide and subsequent modification steps.

Antibiotics have been a critical component of modern medicine since their discovery early in the 20th century, offering effective treatments for otherwise potentially fatal bacterial infections (Fleming, 2001; Gelpi et al., 2015). These “miracle drugs” have also been pivotal to advances in modern medicine by enabling surgeries, organ transplants, chemotherapy, and other procedures requiring immunosuppression. The bacterial ribosome has provided fertile ground in nature and in the research laboratory for antibiotic development and is the target of many antibiotics in clinical use today (Figure 1A). However, resistance to these essential medicines among diverse human bacterial pathogens has now developed against almost all classes of antibiotics in clinical use (Wilson, 2014; Lin et al., 2018). Without action to counter the increasing prevalence of resistance, we face the alternative future of a “post-antibiotic world” where common infections and other diseases once again have a much greater mortality rate (World Health Organization, 2014).

The tuberactinomycins are one example of an important class of ribosome-targeting antibiotics with a long history of clinical use (Anonymous, 1973). Although recently replaced by alternative oral therapies on the World Health Organization (WHO) recommended treatments for active tuberculosis (TB), the tuberactinomycins viomycin and capreomycin remain potent anti-mycobacterial agents. Use of these drugs as second-line treatments was partly designed as a strategy to limit the development of resistance (Bartz et al., 1951; Sutton et al., 1966), but *Mycobacterium tuberculosis* resistance to tuberactinomycins has nonetheless emerged and threatens the efficacy of these antibiotics.

Beginning with their initial discovery and structural characterization in the early 1950s, here, we describe current knowledge on tuberactinomycin biosynthesis, mechanisms of action on the ribosome, and clinically relevant resistance mechanisms that have emerged. Finally, viewed through this lens, we speculate on the future potential use (s) of these and similar antibiotics.

## Chemical structure and biosynthesis of the tuberactinomycin antibiotics

Like many antibiotics in use today, tuberactinomycins are natural products of soil-dwelling bacteria. Viomycin was first isolated from *Streptomyces puniceus* in 1951, and was quickly demonstrated to have potent antibiotic activity against *M. tuberculosis* (Bartz et al., 1951; Finlay et al., 1951). Subsequent discoveries completed the currently recognized tuberactinomycin antibiotic family: tuberactinomycins A, B, and N (also known as enviomycin), and O from *Streptomyces griseoverticillatus* var. *tuberacticus* in 1968; capreomycin from *Streptomyces capreolus* (now *Saccharothrix mutabilis* subspecies *capreolus*) in 1960; and tuberactinamine N, isolated in 1975 (Herr et al., 1960, 1961; Herr and Redstone, 1966; Nagata et al., 1968; Wakamiya et al., 1970; Ando et al., 1971; Yoshioka et al., 1971; Wakamiya and Shiba, 1975). Subsequent work to isolate the individual tuberactinomycins A, B, N, and O revealed that tuberactinomycin B and viomycin were the same compound (Noda et al., 1972). Capreomycin was also initially identified as a mixture of four components (capreomycins IA, IB, IIA, and IIB), which were subsequently isolated and the differences in their core ring substituents characterized (Herr et al., 1960, 1961; Herr and Redstone, 1966; Figure 1B). Notably, however, capreomycin went into clinical use 2 years later (in 1973) as a mixture of all four components (Anonymous, 1973).

Structurally, the tuberactinomycin antibiotics are defined by a conserved cyclic pentapeptide core ring derived from non-ribosomally synthesized pentapeptides, which are cyclized and modified in a series of subsequent reactions to produce the final active compound (Figure 1C). The ~36.3 kb viomycin biosynthesis gene cluster in *Streptomyces vinaceus* contains 20 open reading frames (ORFs) encoding all components necessary for the biosynthesis, regulation, export, and activation of viomycin (Thomas et al., 2003; Barkei et al., 2009). First, the cyclic pentapeptide core (common to all tuberactinomycins) is assembled by VioA, VioI, VioF, and VioG using at least one L-serine and at least one of the non-proteinogenic amino acids 2,3-diaminopropionate, L-capreomycidine, and β-ureidodehydroalanine. VioJ then desaturates part of the ring and additional modifications are added by VioL (carbamoylation), VioM/VioO (N-acylation), and VioQ (hydroxylation). Other genes within the biosynthetic cluster are not directly associated with the building of viomycin, but perform other essential steps such as the synthesis of specialized amino acid building blocks (VioP). The cluster also includes a viomycin resistance gene (*vph*, encoding viomycin phosphotransferase) to protect the producing bacterium from self-intoxication.

The capreomycin biosynthetic gene cluster in *S. mutabilis* subspecies *capreolus* has also been characterized and comprises 33 ORFs (Felnagle et al., 2007). Of these 33 ORFs, only 19 are proposed to be involved with the production of capreomycin, while the function of the other 14 ORFs remains unknown. These ORFs have no sequence similarity to genes that suggest an obvious role in capreomycin biosynthesis and sequence analyses of the viomycin and capreomycin gene clusters show that homology exists only between the already-identified ORFs (Felnagle et al., 2007). The capreomycin biosynthesis gene cluster encodes three different resistance enzymes: *cph* (the direct homolog of viomycin phosphotransferase *vph*), *cac* (a putative capreomycin acetyltransferase), and *cmnU* (proposed to encode a 16S rRNA m^1^A1408 aminoglycoside-resistance methyltransferase; Skinner and Cundliffe, 1980; Thiara and Cundliffe, 1995). *cac* is required in addition to *cph,* as the latter enzyme modifies a hydroxyl group present in capreomycin IA and IIA but absent in IB and IIB; *cac* is proposed to modify an amino group common to all four capreomycin molecules. Why the capreomycin biosynthesis cluster also encodes the rRNA modification enzyme CmnU is less clear, though it is possible that this additional target-based resistance mechanism alleviates residual toxicity in the modified capreomycin product of *cph* or *cac* or, alternatively, against a currently unknown secondary metabolite that is produced in the process of capreomycin but not viomycin biosynthesis.

## 70S ribosome binding and tuberactinomycin mechanism of action

Tuberactinomycins inhibit the process of bacterial translation, i.e., protein synthesis by the ribosome. Specifically, the bactericidal effect of this class of antibiotics is derived from their capacity to block the process of translocation, or movement of the mRNA-tRNA pairs following peptide bond formation to position the next three-nucleotide codon within the aminoacyl tRNA binding site (A site) for decoding (Modolfll and Vazquez, 1977). During this step, the deacylated tRNA is moved from the peptidyl tRNA site (P site) to the exit site (E site), while the A-site tRNA, now carrying the nascent polypeptide chain, is moved to the P site. Normally, the movement of the tRNAs is driven by elongation factor G (EF-G) and hydrolysis of GTP. Viomycin binds the ribosome in the pre-translocation state, stabilizing 16S nucleotides A1492 and A1493 in their active conformation and preventing the backward movement of the 30S body and head domain (Holm et al., 2019; Belardinelli et al., 2021). Viomycin has been shown to prevent translocation for a minimum of ~45 s (longer with higher viomycin concentration), thereby inhibiting overall translation and hindering essential cell processes (Holm et al., 2016). While a detailed discussion is beyond the scope of this focused mini-review, it is also noteworthy that the tuberactinomycins have overlapping binding sites (see Figure 1), mechanisms of action, and susceptibility to bacterial resistance mechanisms with other ribosome-targeting antibiotics (Maus et al., 2005; Wilson, 2014; Lin et al., 2018; Holm et al., 2019).

Optimal tuberactinomycin binding to the 70S ribosome, and thus the anti-mycobacterial activity of these antibiotics, is dependent on intrinsic ribose 2’-OH methylation incorporated by a single Class I S-adenosyl-L-methionine (SAM)-dependent housekeeping methyltransferase TlyA, encoded by the *tlyA* gene. Two subfamilies of TlyA are known, TlyA^I^ which methylates 23S rRNA nucleotide C1920 (*E. coli* numbering; C2158 in *M. tuberculosis*) and dual specificity TlyA^II^ which methylates C1920 and 16S rRNA nucleotide C1409 (C1402 in *M. tuberculosis*; Johansen et al., 2006; Monshupanee et al., 2012). TlyA^II^ is conserved in mycobacteria, while TlyA^I^ is possessed by other diverse bacterial species, including *Thermus thermophilus* which is used extensively in ribosome structural studies, but not in *E. coli*. As a result, *E. coli* is intrinsically less susceptible to tuberactinomycins (Johansen et al., 2006; Monshupanee et al., 2012), and *M. tuberculosis* clinical resistance can arise through loss of TlyA activity, as discussed further below.

**Figure 2:**
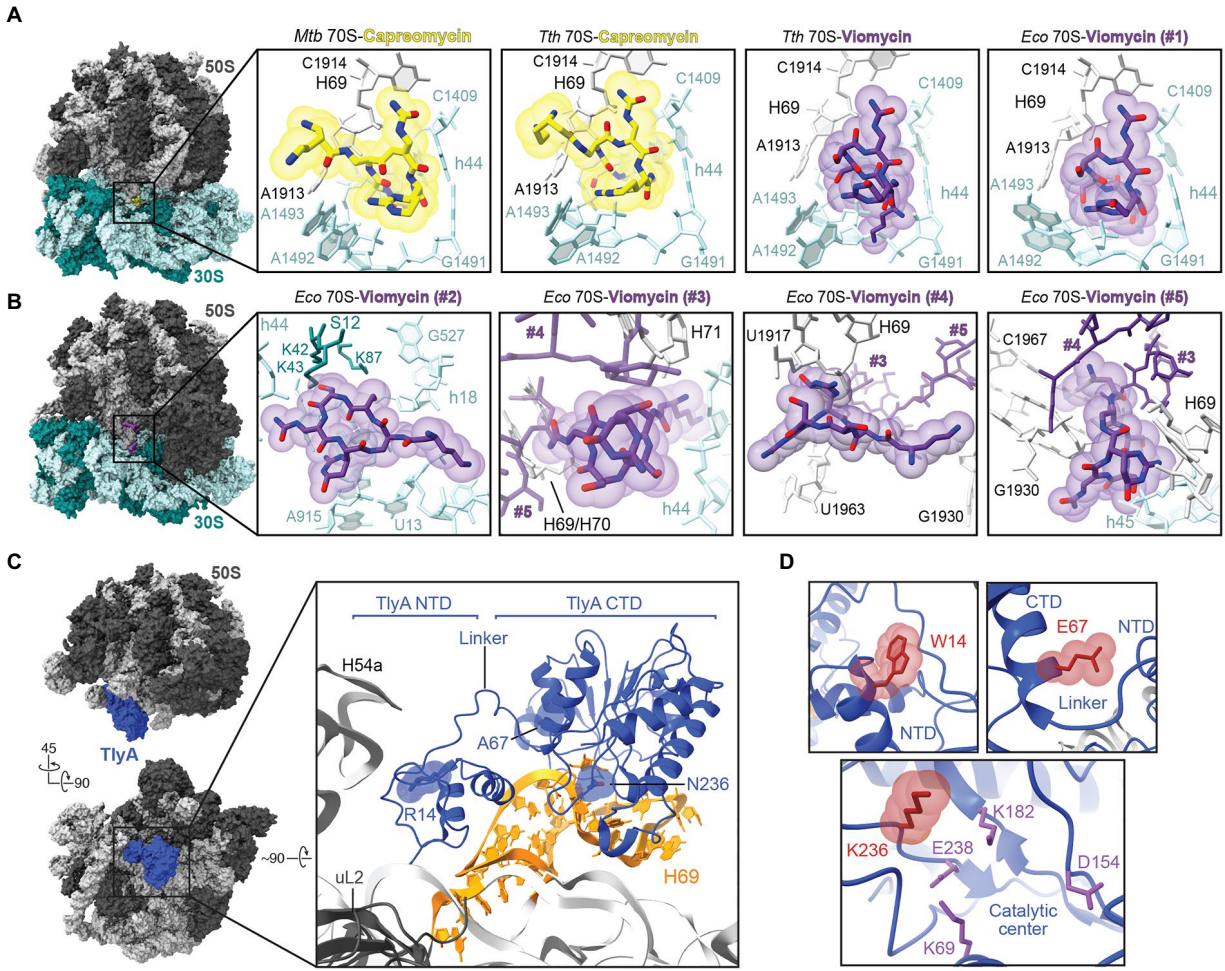
Molecular basis of tuberactinomycin action and resistance. **(A)** Overview of the *Mycobacterium tuberculosis (Mtb)* 70S structure (PDB code 5 V93) with the capreomycin (yellow) binding site indicated within the boxed region. Zoomed-in views are also shown of the primary tuberactinomycin antibiotic binding site at the 30S and 50S interface for (*left* to *right*): capreomycin bound to the *M. tuberculosis* 70S (PDB code 5 V93) and *T. thermophilus* (*Tth*) 70S (PDB code 4V7M), and for viomycin (purple) bound to *T. thermophilus* 70S (PDB code 4V7L) and *E. coli* (*Eco*) 70S (PDB code 6LKQ). For ease of comparison, nucleotides are labeled with *E. coli* numbering where shown. **(B)** Overview of the *E. coli* 70S-viomycin complex and zoomed-in views of the four additional viomycin binding sites, #2 to #5, identified in this structure (PDB code 6LKQ). Note that viomycin molecules at sites #3-#5 make interactions with each other and are shown in each view but with only the focused viomycin shown with atom coloring. **(C)** Two views of the structure of the mycobacterial 50S subunit-TlyA complex (*left*). In the top view, the 50S subunit is shown in the same orientation as in the 70S ribosome views of *panels A* and *B*. Also highlighted in the zoomed-in view are TlyA (blue), its rRNA binding site on H69, and three amino acid substitutions leading to loss of TlyA activity from missense *tlyA* mutations in *M tuberculosis* clinical isolates. **(*D*)** Zoomed-in views of the sites of amino acid substitution shown with the residue arising from clinical resistance mutation (red).

Structures of viomycin and capreomycin bound to 70S ribosomes from several bacterial species have been determined via X-ray crystallography or cryogenic electron microscopy (cryo-EM) and these antibiotics share a common binding site located primarily in the ribosome 30S subunit decoding center and contacting the adjacent 30S/50S subunit interface between 16S rRNA helix 44 (h44) and 23S rRNA Helix 69 (H69; Stanley et al., 2010; Yang et al., 2017; Figure 2A). This binding site is largely conserved when either capreomycin or viomycin are bound to ribosomes from different bacterial species. Additionally, both drugs bind to the ribosome in a very similar manner whether the 70S complex is in the classical or rotated state (i.e., the relative orientation of the subunits to each other at different points in a cycle of translation; Stanley et al., 2010; Yang et al., 2017; Zhang et al., 2020). Structural analyses of the *E. coli* 70S-viomycin complex identified four additional sites: one exclusively on the 30S subunit near ribosomal protein S12 (Figure 2B; Vio#2) when the ribosome is in either a classical or rotated state, and a triple cluster at a distinct inter-subunit site which appears unique to the rotated state (Figure 2B; Vio#3 to #5; Zhang et al., 2020).

At the common site on the 30S shared by capreomycin and viomycin, antibiotic binding affects the positioning of 16S rRNA nucleotides A1492 and A1493, and 23S rRNA nucleotide A1913 (*E. coli* numbering), which surround the A-site tRNA in the ribosomal decoding center. Flipping of A1492 and A1493 from h44 is established as a mechanism by which the ribosome interrogates the mRNA-tRNA pairing by contacting the minor groove of the codon-anticodon pairing (Ogle et al., 2001). Together with repositioning of A1913 to hydrogen bond with the A-site tRNA, the interactions of these nucleotides likely result in the observed increased tRNA affinity for the A site, hindering of subunit movement during translocation, and promotion of back translocation in the presence of tuberactinomycins (Peske et al., 2004; Szaflarski et al., 2008; Holm et al., 2016, 2019; Belardinelli et al., 2021). Consistent with this site of action for the tuberactinomycins, selection of capreomycin resistance in *T. thermophilus* identified mutations or deletions at A1913 and the adjacent U1915 at the tip of H69 (Monshupanee et al., 2008). Further, these rRNA changes did not affect C1920 modification by TlyA suggesting a direct effect on the drug binding site structure.

While binding at the common site (Figure 2A; Vio#1) can explain much of the observed action of viomycin and is supported by the above noted mutational analyses, identification of additional binding sites for this drug (though, to date, not for capreomycin; Figure 2B; Vio#2–5) open the possibility of additional contributions to its mechanism of action. As noted by Zhang et al. in their report on the 70S-viomycin complex, identification of essentially equivalent viomycin locations in the A site in both the classical and rotated state calls into question the role of drug binding at this site in hindering translocation (Zhang et al., 2020). Alternatively, the triple cluster of viomycin molecules at the subunit interface may be the origin of inhibition of subunit dissociation and inter-subunit movement, as it is observed only in the rotated state (Figure 2B). This possibility is supported by earlier single molecule studies suggesting multiple binding sites (Feldman et al., 2010), and the proximity of one of the three viomycins (Vio#3) to the C1920 ribose which is modified by TlyA. However, the high concentration of viomycin (0.5 mM)—which may be necessary to form the unusual triple cluster of drug molecules—and the absence of TlyA-encoded modifications at C1409 and C1920 in the *E. coli* ribosomes used for these studies, leave some uncertainty over the contribution of these binding sites to viomycin action. On the other hand, the previously identified common binding site (Figures 2A,B; Vio#1) cannot readily explain the specific dependence of viomycin and capreomycin activity on C1920 methylation distant from this site. The influence of ribose methylation on C1920 sugar pucker and H69 helical structure influencing the architecture of the common binding at the tip of H69 site has recently been proposed (Laughlin et al., 2022), but further studies are needed to tease apart these important molecular details of tuberactinomycin action on the ribosome.

## Clinical use of tuberactinomycins and mechanisms of resistance

According to the WHO, TB was the second-leading cause of death from a single infectious agent worldwide in 2020 (behind COVID-19), resulting in approximately 1.5 million deaths (World Health Organization, 2021). Further, of an estimated 9.9 million individuals who developed TB in 2020, 7.5% of those tested for drug resistance indicated infection with either a multi (MDR)-, pre-extensively, or extensively drug-resistant strain of *M. tuberculosis*. Tuberactinomycins, and capreomycin in particular, have a long history in the treatment of these drug-resistant cases of TB (World Health Organization, 2016), with its inclusion on the WHO Model List of Essential Medicines reflecting this global importance (World Health Organization, 2017). However, these drugs suffer side effects, including ototoxicity and nephrotoxicity, and were thus not recommended for use in children or adult patients with mild forms of TB (World Health Organization, 2016). Additionally, as noted earlier, with the wider availability of all-oral treatments, the WHO revised guidelines around the treatment of MDR-TB in 2018, and currently recommends against the use of capreomycin (which is an injectable agent; World Health Organization, 2018). However, with their retained anti-mycobacterial efficacy, the tuberactinomycins may still play an important role in the treatment of TB or other bacterial infectious diseases, particularly if limitations due to side effects can be overcome with new generations of drugs. As such, defining the basis of resistance mechanisms which have already emerged through clinical use of these drugs will also be an important future consideration.

Tuberactinomycins bind at a similar location on the ribosome to the aminoglycosides kanamycin and amikacin, which have also been used as second-line drugs for the treatment of TB, and cross-resistance between these drug classes in *M. tuberculosis* has been observed (Maus et al., 2005; Jugheli et al., 2009; Akbergenov et al., 2011). Clinical isolates of tuberactinomycin-resistant *M. tuberculosis* typically arise through mutation of the genes encoding either rRNA or TlyA. Sites of resistance mutations in 16S rRNA cluster around the drug binding site at the subunit interface (Figure 2A) and include the TlyA target nucleotide in h44 (C1409), its base pairing partner (G1491), and several other nearby residues in the ribosome decoding center (Maus et al., 2005; Akbergenov et al., 2011; Phelan et al., 2019; Olawoye et al., 2021). Deletion of 23S rRNA nucleotide A1916 in the loop of H69 has also been observed in a capreomycin-resistance clinical *M. tuberculosis* isolate (Johansen et al., 2006).

Mutations in *tlyA* leading to clinical resistance include both nonsense (premature stop codon) and missense mutations that result in amino acid substitutions that eliminate TlyA enzymatic activity (Johansen et al., 2006; Monshupanee et al., 2012; Li et al., 2019; Phelan et al., 2019). The TlyA structure comprises an amino-terminal domain (NTD), which adopts a ribosomal protein S4-like fold, and a Class I methyltransferase carboxyl-terminal domain (CTD), connected by a short linker which is important for cosubstrate SAM binding within the CTD (Witek et al., 2017). Recent structural studies have elucidated both the full-length structure of TlyA and the molecular basis for recognition and modification of one of its two substrates, C1920 on the 50S subunit (Figure 2C; Laughlin et al., 2022). TlyA interaction with the 50S subunit exclusively exploits contacts with 23S rRNA at H69 and the adjacent rRNA junction, via a contiguous surface spanning both protein domains which is enriched in basic residues. The NTD appears critical for correct substrate recognition as substitution of either of two conserved arginine residues (Arg6 and Arg20), distant from the enzyme active site, eliminates C1920 ribose methylation. These residues act in concert to recognize the unique 23S rRNA structure at the base of H69. Similarly, critical residues were also identified in the CTD, including Phe157 which appears to stabilize a “flipped” conformation of C1920 for optimal orientation of the ribose 2’-OH for modification. Mutations in *tlyA* that inactivate TlyA and lead to clinical capreomycin resistance have been identified in both protein domains and can be rationalized based on these recent structural insights (Figure 2D). For example, a R14W substitution (Phelan et al., 2019) likely results in capreomycin resistance by disruption of the NTD structure and thus critical contacts to 23S rRNA made by the nearby Arg6 and Arg20. Another common mutation found in resistant *M. tuberculosis* results in a lysine substitution of Gln236 in the TlyA CTD (Walker et al., 2018); this residue immediately follows a short loop that envelops the flipped C1920 base and additionally results in placement of a basic side chain close to residue Glu238 which has been proposed to play an important role in catalysis (Figure 2D; Arenas et al., 2011). Finally, a third clinical capreomycin-resistance mutation was identified which results in an A67E substitution at a site distant from both the catalytic center and NTD region critical for rRNA (Phelan et al., 2019; Laughlin et al., 2022). How this change impacts TlyA function is less clear, but the larger charged residue would likely disrupt a hydrophobic pocket occupied by Trp62 of the TlyA inter-domain linker (Figure 2D), which was previously shown to be important for SAM binding and which could also influence correct NTD/CTD association or inter-domain communication during substrate recognition (Witek et al., 2017; Laughlin et al., 2022).

While these recent studies have elucidated much about TlyA’s mechanism of action and the basis for its inactivation by mutations in drug-resistant *M. tuberculosis*, several open questions remain. In the case of dual specificity enzymes (TlyA^II^) like that of *M. tuberculosis*, how the enzyme adapts to recognize and modify its target nucleotide in the 30S is currently not known in detail. However, a significantly overlapping molecular surface of the TlyA NTD and CTD is again critical, but with distinct dependencies on functionally critical residues compared to 50S subunit modification (Laughlin et al., 2022). In terms of clinical resistance, a broader question is why *M. tuberculosis* and many other bacteria maintain TlyA given its contribution to tuberactinomycin activity on the ribosome. Removal of endogenous TlyA activity appears to incur little cost to fitness, but may result in a reduction of functional 70S ribosomes (Salamaszynska-Guz et al., 2014; Freihofer et al., 2016). Identification of a critical function for TlyA—perhaps unrelated to rRNA modification—or conditions under which it is revealed will require further careful investigation.

## Conclusion and future perspectives

Tuberactinomycins are a valuable class of ribosome-targeting antibiotics that have played an important role in treating drug-resistant TB. Despite current recommendations against their use, retained tuberactinomycin efficacy leaves open the possibility for future applications, especially if new derivatives can be obtained which counter the emerging resistance mechanisms and overcome toxicity issues. Previous studies have demonstrated that the addition of various compounds to cultures of bacteria expressing the biosynthetic gene cluster can alter the products of antibiotic production resulting in new compounds based on the tuberactinomycin core (Morse et al., 1997). Additionally, engineering of the biosynthetic cluster or alteration of supplied building blocks could provide starting points for further evolution of this drug family using semi-synthetic chemical approaches. In addition to providing new alternatives to treat TB, an expanded tuberactinomycin family would likely find application in the fight against other antibiotic-resistant bacterial infections. For example, viomycin has been shown to be effective vancomycin-resistant enterococci and MRSA (Dirlam et al., 1997; Linde et al., 1997). Additionally, capreomycin has been shown in cell culture to have anti-viral activity against SARS-CoV2 despite its previous use as strictly an antibiotic (Kumar et al., 2021). Combined with efforts to reduce unwanted side effects, future efforts to expand the tuberactinomycin drug class may offer a fruitful avenue to much-needed treatments for infectious diseases.

## Author contributions

ZL conducted a review of relevant literature and prepared a first draft of the manuscript. ZL and GC wrote sections of the final manuscript. Both authors contributed to the article and approved the submitted version.

## Funding

Our research on TlyA is supported by NIH/NIAID awards T32-AI106699 (to ZL) and R01-AI088025 (to GC).

## Conflict of interest

The authors declare that the research was conducted in the absence of any commercial or financial relationships that could be construed as a potential conflict of interest.

## Publisher’s note

All claims expressed in this article are solely those of the authors and do not necessarily represent those of their affiliated organizations, or those of the publisher, the editors and the reviewers. Any product that may be evaluated in this article, or claim that may be made by its manufacturer, is not guaranteed or endorsed by the publisher.
